# Supplementary material for: Electrofreezing of liquid water at ambient conditions
Source: Nat Commun. 2024 Feb 29;15:1856. doi: 10.1038/s41467-024-46131-z (PMC10904787; doi:10.1038/s41467-024-46131-z)
Supplement: Supplementary file 1 — Supplementary Information [file 41467_2024_46131_MOESM1_ESM.pdf]

Supplementary Information Electrofreezing of Liquid Water at Ambient Conditions

Giuseppe Cassone[1*] and Fausto Martelli[2,3*]

[1]Institute for Chemical-Physical Processes, National Research Council, Viale F. Stagno d'Alcontres 37, Messina, 98158, Italy.
[2]IBM Research Europe, Keckwik Lane, Daresbury, WA4 4AD, United Kingdom.
[3]Department of Chemical Engineering, University of Manchester, Oxford Road, Manchester, M13 9PL, United Kingdom.

*Corresponding author(s). E-mail(s): cassone@ipcf.cnr.it; fausto.martelli@ibm.com;

Supplementary Results

Infrared spectra shown in Fig. 1 of the main text have been determined using the software TRAVIS [1, 2] from the centers of the Maximally Localised Wannier Functions (MLWFs) [3, 4] calculated on the fly during the *ab initio* molecular dynamics (AIMD) simulations. Molecular dipoles from MLWFs centers can be determined as:

$$\mu = -2e\sum_i \mathbf{r}_i + e\sum_j Z_j \mathbf{R}_j\,, \tag{1}$$

where e is the electron charge, $\mathbf{r}_i$ is the position vector of the MLWF center i, Z_j is the atomic number of the nuclei j whilst $\mathbf{R}_j$ is the position vector of this latter. This way, the IR spectra at the investigated field intensities were computed as the Fourier transform of the molecular dipole autocorrelation function along the last 50 ps of the respective simulation trajectories.

To track molecular reorientations under the field action, we compute the distributions of the angle θ formed between the instantaneous water molecular dipole vectors and the field direction (i.e., z-axis), Fig. Supplementary1. Interestingly, whilst the field is capable of reorienting a large fraction of water dipoles already at 0.05 VÅ^{-1}, the electrostatic potential gradient producing this field strength does not induce a

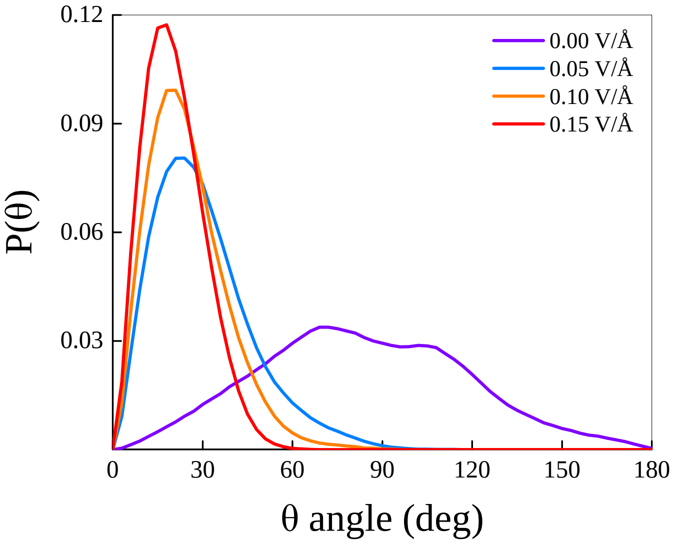

Fig. Supplementary 1 Distributions of the θ angle formed between the instantaneous molecular dipole vectors during the last 50 ps of the respective trajectories and the EF direction for diverse field intensities applied along the z-axis.

net suppression of the translational degrees of freedom of the molecules, as shown in Fig. 3 of the main text. The enhancement of the water dipoles at increasingly high fields is also visible from the dipole distributions reported in Fig. Supplementary2-a, showing a progressive shift towards larger magnitudes and a slight narrowing of the distributions.

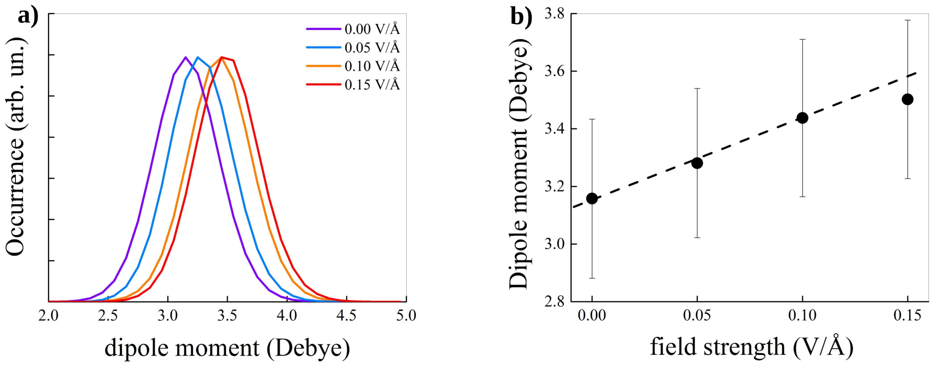

Fig. Supplementary 2 (a) Distributions of the magnitude of the water dipoles extracted from the last 50 ps of the respective simulations at different field intensities determined from the MLWFs centers. (b) Average water dipole and associated standard deviation extracted from the distributions in (a). It is noteworthy to point out the interruption of the linear response regime for field strengths producing water electrofreezing.

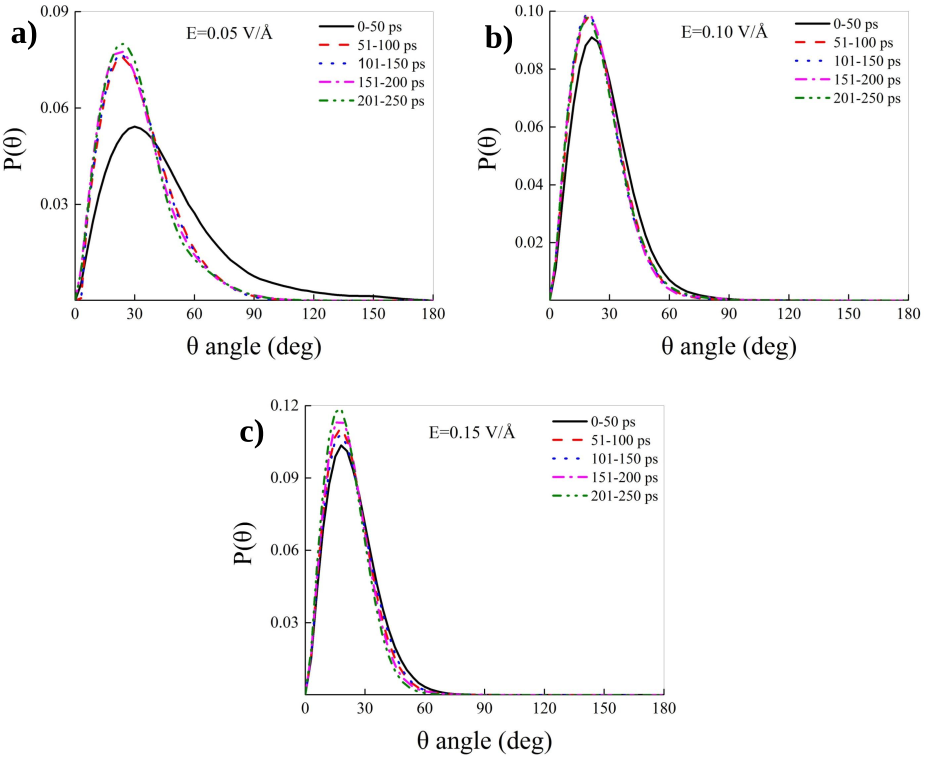

Fig. Supplementary 3 Distributions of the θ angle at 0.05 VÅ^{-1} (a), 0.10 VÅ^{-1} (b), and 0.15 VÅ^{-1} (c) measured at consecutive time windows. The black solid curves refer to the first 50 ps, dashed red lines to the time window $51-100$ ps, dotted blue curves to the time window $101-150$ ps, the dashed-dotted magenta lines to the time window $151-200$ ps, and the dashed-dotted-dotted green curves to the window $201-250$ ps.

Fig. Supplementary2-b reports the profile of the dipole moment with the field strength. It is possible to recognize a linear regime holding up to a strength of 0.10 VÅ^{-1}. Thus, the transition from the liquid to the f-GW phase is also marked by the breakdown of the linear response regime to external electric fields (EFs). Additionally to this analysis, it is worth monitoring the temporal dependence of the P(θ) distributions at disjoint time windows, a procedure allowing for disclosing the dynamical response of the sample. As reported in Fig. Supplementary3, the field-induced reorientation of the molecular dipoles takes place on fast timescales and achieves saturation within the first 50 ps of dynamics at all field intensities, except for the weakest field (Fig. S3-a), where nonetheless the convergence of the dipolar response is reached in less than 100 ps. Our results are in qualitative agreement with Ref. [5].

Fig. Supplementary4 reports the oxygen-oxygen radial distribution functions computed at consecutive, disjoint time windows of 50 ps. At 0.05 VÅ^{-1}, the $g_{OO}(r)$ converges to a steady profile after 50 ps (Fig. Supplementary4-a), while convergence

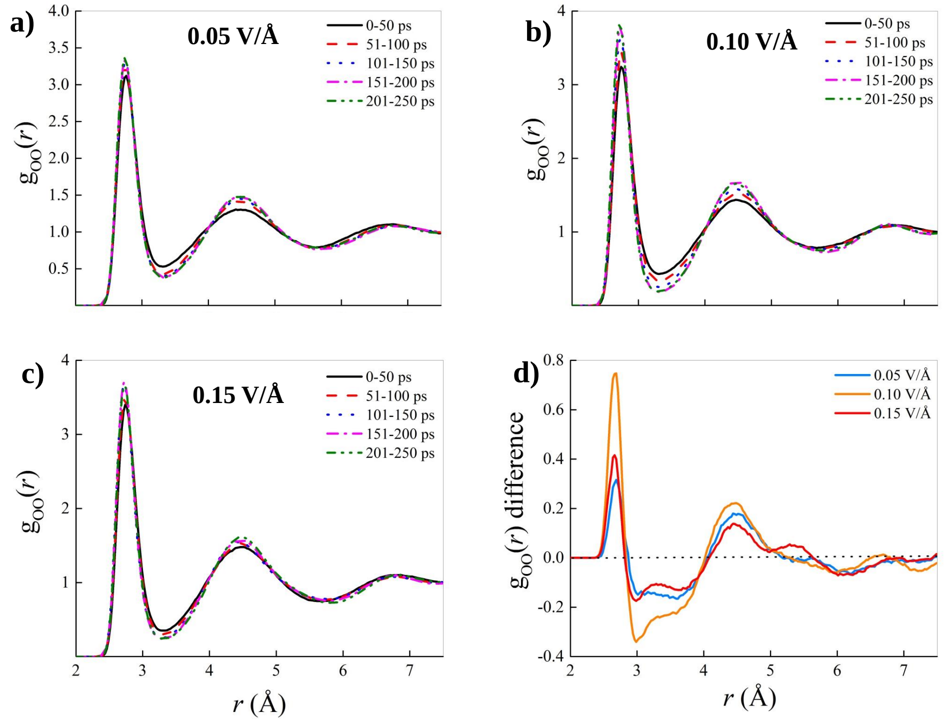

Fig. Supplementary 4 Oxygen-oxygen radial distribution functions determined for disjoint time frames of 50 ps each determined from the *ab initio* molecular dynamics simulations conducted at 0.05 VÅ^{-1} (a), 0.10 VÅ^{-1} (b), and 0.15 VÅ^{-1} (c) field strengths. The black solid curves refer to the first 50 ps, dashed red lines to the time window $51-100$ ps, dotted blue curves to the time window $101-150$ ps, the dashed-dotted magenta lines to the time window $151-200$ ps, and the dashed-dotted-dotted green curves to the window $201-250$ ps. In panel (d), the point-by-point difference of the oxygen-oxygen radial distribution functions determined in the final and the initial time frames (i.e., during the last 50 and first 50 ps, respectively) for diverse field intensities is shown.

is achieved only after 150 ps for 0.10 VÅ^{-1} (Fig. Supplementary4-b) and 200 ps for 0.15 VÅ^{-1} (Fig. Supplementary4-c). The $g_{OO}(r)$ computed within the last $50-100$ ps for 0.10 VÅ^{-1} and 0.15 VÅ^{-1} resemble the $g_{OO}(r)$ of a low-density amorphous (LDA) (see main text). To shed some light on the dynamical reorganization of the water structure induced by the external field, we have evaluated the oxygen-oxygen radial distribution function differences between the last 50 ps and the first 50 ps time frames of each simulation, as reported in Fig. Supplementary4-d. Whereas at 0.05 VÅ^{-1} structural differences between the initial and the final time windows appear to be small – as also visible in Fig. Supplementary4-a –, a field of intensity equal to 0.10 VÅ^{-1} induces much larger global reorganizations towards more structured molecular correlations in the system (Fig. Supplementary4-d, yellow curve). On the other hand, the evidence that these differences are smaller in the sample exposed to a 0.15 VÅ^{-1} field (Fig. Supplementary4-d, red curve) has to be ascribed to a faster initial reorganization

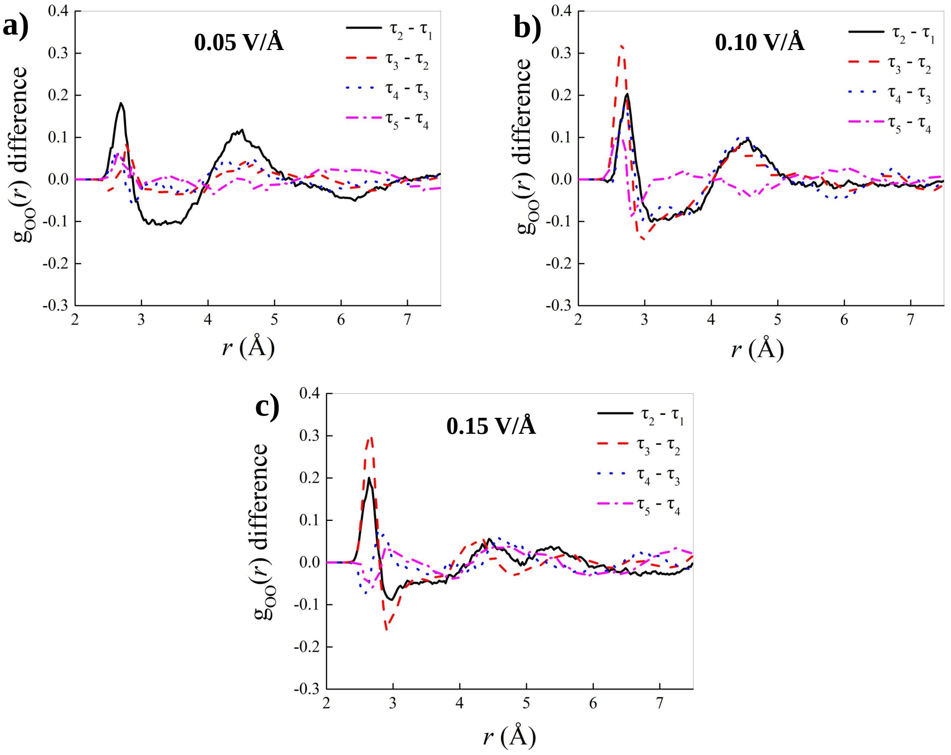

Fig. Supplementary 5 Point-by-point difference between the oxygen-oxygen radial distribution functions (OO-RDFs) determined for disjoint consecutive time frames of 50 ps and presented in Fig. S4, each conducted at 0.05 VÅ^{-1} (a), 0.10 VÅ^{-1} (b), and 0.15 VÅ^{-1} (c). Black solid curves refer to the difference of the OO-RDF calculated over the $51-100$ ps time-frame and that evaluated over the $1-50$ ps time-window. Dashed red lines refer to the difference of the OO-RDF calculated over the $101-150$ ps time-frame and that evaluated over the $51-100$ ps time-window. Dotted blue curves refer to the difference of the OO-RDF calculated over the $151-200$ ps time-frame and that evaluated over the $101-150$ ps time-window. Dashed-dotted magenta lines refer to the difference of the OO-RDF calculated over the $201-250$ ps time-frame and that evaluated over the $151-200$ ps time-window.

taking place since the first 50 ps of dynamics, whereas longer timescales ($\sim$ 200 ps) are somehow needed for bringing to completion the structural transition in the simulated sample, as shown in Fig. Supplementary4-c.

Fig. Supplementary5 shows the point-by-point difference of the oxygen-oxygen radial distribution functions displayed in Fig. Supplementary4 calculated between adjacent time-windows τ. This way, the 1st window ($\tau_2 - \tau_1$, solid black curves) is the difference between the oxygen-oxygen radial distribution function calculated over the $51-100$ ps time-frame and that evaluated over the $1-50$ ps time-window. Similarly, the 2nd window ($\tau_3 - \tau_2$, dashed red lines) is the difference between the $g_{OO}(r)$ measured during the $101-150$ ps time-frame and that sampled during the previous $51-100$ ps time-window, and so on and so forth for the 3rd ($\tau_4 - \tau_3$, dotted

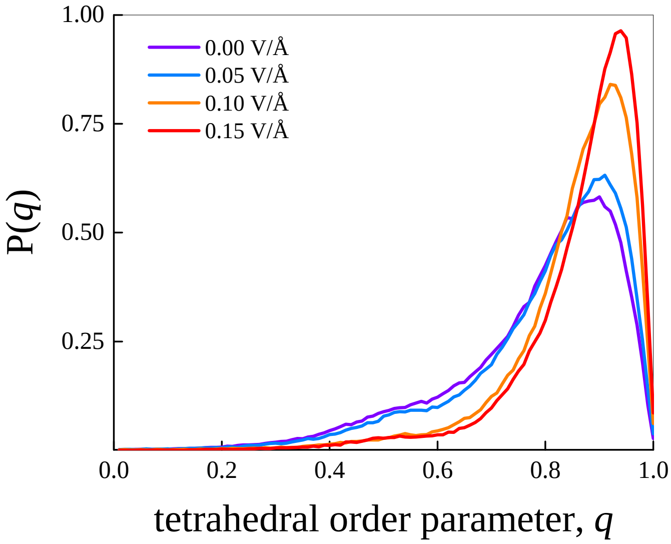

Fig. Supplementary 6 Distribution of the orientational tetrahedral order parameter q determined at zero field (violet curve) and at field strengths equal to 0.05 VÅ^{-1} (blue curve), 0.10 VÅ^{-1} (orange curve), and 0.15 VÅ^{-1} (red curve).

blue curves) and the 4th ($\tau_5 - \tau_4$, dashed-dotted magenta lines) windows (see also the caption of Fig. Supplementary5). Whereas structural equilibrium is reached after $\sim 50 - 100$ ps in presence of an external electric field having intensity of 0.05 VÅ^{-1} (Fig. Supplementary5-a), longer timescales are needed when stronger fields are applied. In fact, the large amplitude $g_{OO}(r)$ differences recorded in the 2nd windows for fields of 0.10 VÅ^{-1} (Fig. Supplementary5-b) and 0.15 VÅ^{-1} (Fig. Supplementary5-c) witness that significant structural modifications are triggered by the external field at timescales of $\sim 100 - 150$ ps. Consistently with the previous analyses, although long times (i.e., $\sim 200 - 250$ ps) are needed to almost fully damp structural fluctuations between consecutive time-windows at the intermediate field of 0.10 VÅ^{-1}, the larger field strength of 0.15 VÅ^{-1} drives the system towards a structural (near-)equilibrium within timescales on the order of $\sim 150 - 200$ ps, as shown in Fig. Supplementary5-c.

In Fig. Supplementary6 we report $P(q)$, the distribution of the tetrahedral order parameter q defined as

$$q = 1 - \frac{3}{8} \sum_{j=1}^{3} \sum_{k=j+1}^{4} \left(\cos \psi_{jk} + \frac{1}{3} \right)^2 \tag{2}$$

where ψ_{jk} is the angle formed between the oxygen atoms of the water molecule under consideration and its nearest neighbour oxygen atoms j and k. The tetrahedral order parameter q was originally proposed by Chau and Hardwick [6] and subsequently rescaled by Errington and Debenedetti [7] so that the average value of q varies from 0

for an ideal gas to 1 for a regular tetrahedron. From Fig. Supplementary6 it is possible to observe that the samples in the absence of a field and in the presence of a field of 0.05 VÅ^{-1} show a very similar tetrahedral character. A major change occurs at stronger fields signaling the transition to the more ordered f-GW phase.

Similar conclusions can be drawn upon inspecting the local structure index (LSI) [8, 9], an insightful order parameter that can be employed to characterize the LDL and HDL molecular environments and defined as the inhomogeneity on the distribution of radial distances

$$I = \frac{1}{N}\sum_{j=1}^{N}\left[\Delta_{j+1,j} - \langle\Delta\rangle\right]^2 \tag{3}$$

where $\Delta_{j+1,j} = r_{j+1} - r_j$ is the distance between particles within a cutoff distance of 3.7 Å from a reference molecule and $\langle\Delta\rangle$ is the average overall neighbours of a molecule within the given cutoff. The LSI, therefore, provides a convenient quantitative measure of the fluctuations in the distance distribution surrounding a given water molecule within a sphere defined by a radius of 3.7 Å. In doing so, the index I measures the extent to which a given water molecule is surrounded by well-defined first and second coordination shells. In Fig. Supplementary7, we report the LSI computed for the three EFs here inspected at time windows of 50 ps. It is possible to observe the development of hints of a bimodal distribution in the cases of 0.10 VÅ^{-1} and 0.15 VÅ^{-1} in correspondence with the transition to f-GW. This can be also appreciated from the lower panel of Fig. Supplementary7, reporting the LSI computed in the time window $[201 - 250]$ ps for 0.15VÅ^{-1} and for the LDA simulated via classical molecular dynamics at $T = 200$ K. The latter has been obtained upon quenching liquid water from $T = 300$ K to $T = 200$ K at a quenching rate of 1 K/ns, as reported in Refs. [10–13]

Somewhat related to the local and global degree of order of the H-bond network, is its kinetics. In particular, we performed a structural analysis of the H-bond network and identified a H-bond through the following geometric conditions (that must be simultaneously fulfilled): two water molecules are considered as H-bonded if $R^{(OO)} \leq$ 3.5 Å and $\angle$O-H$\cdots$O$\leq 30°$, where R^{OO} is the instantaneous distance between the oxygen atoms. From this, we calculated the time autocorrelation function of H-bonds as:

$$c(t) = \frac{\sum_{\langle i,j\rangle} s_{ij}(t_0)s_{ij}(t_0+t)}{\sum_{\langle i,j\rangle} s_{ij}(t_0)}\,, \tag{4}$$

where the indices i and j run on all pairs of first-neighbour molecules which at t_0 were H-bonded, t_0 being the time at which the measurement process begins; $s_{ij} = 1$ if the criterion for the presence of a H-bond is fulfilled, $s_{ij} = 0$ otherwise. The results were averaged over hundreds of initial configurations. Fig. Supplementary8 shows the continuous (Fig. Supplementary8-a) and intermittent (Fig. Supplementary8-b) autocorrelation functions $c(t)$ of the H-bonds for different field intensities. Within the intermittent definition of $c(t)$, a given H-bond is allowed to cleave within timescales ≤ 5 fs to account for bond fluctuations. Thus, within this latter fast timescale, we always assign to s_{ij} a value equal to 1 when considering the intermittent autocorrelation function (see eq. (4)). The application of a 0.05 VÅ^{-1} field induces only a relatively moderate – with respect to the zero-field case – slow down of the dynamics

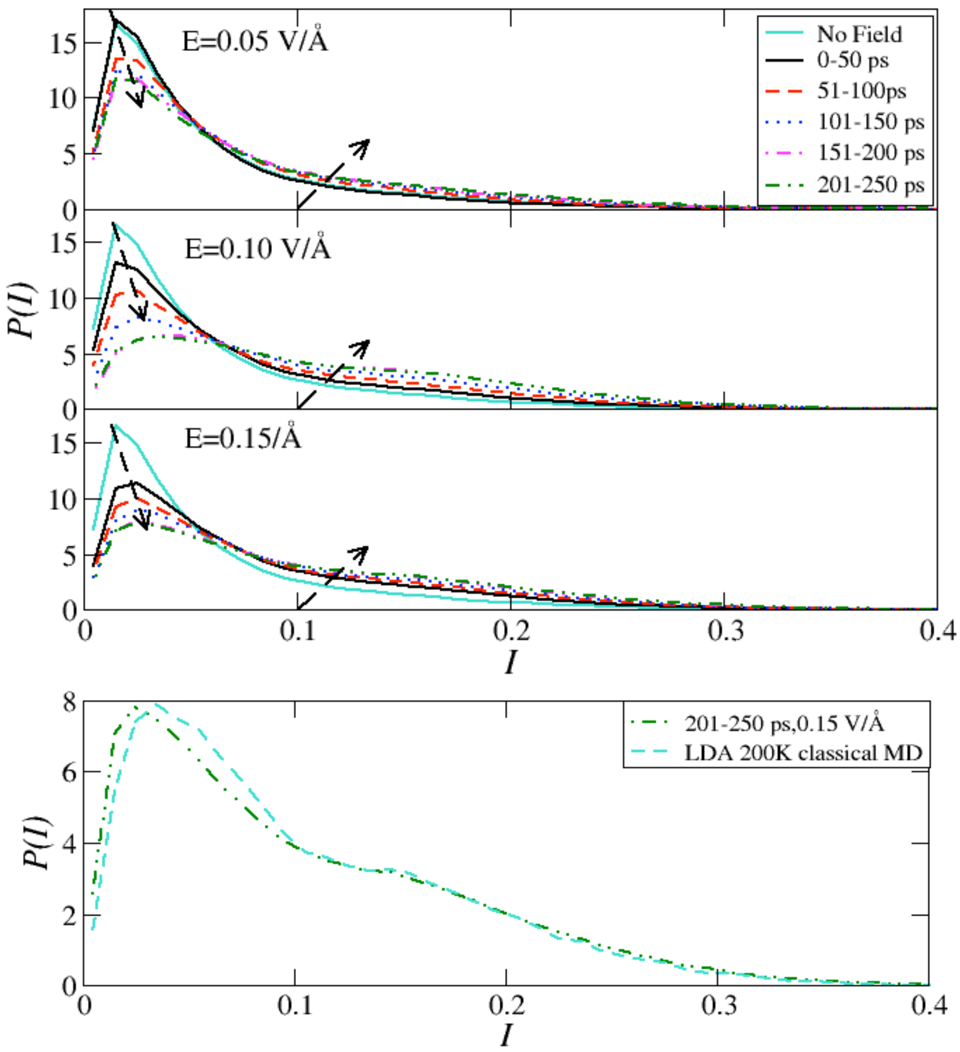

Fig. Supplementary 7 Upper panel: $P(I)$ computed for the case at 0.05 VÅ^{-1}, 0.10 VÅ^{-1}, and 0.15 VÅ^{-1} at disjoint time windows of 50 ps. Lower panel: comparison between the LSI computed for 0.15VÅ^{-1} and the LSI computed for LDA at $T = 200$ K from classical molecular dynamics.

of the H-bond network recorded by means of the continuous $c(t)$ function (Fig. Supplementary8-a). Instead, significantly more drastic effects are recorded upon applying fields of 0.10 and 0.15 VÅ^{-1}. Interestingly, the changes produced on the H-bond network kinetics by these field regimes qualitatively resemble those induced by a sizable ($\sim$ 40 K) decrease of the temperature [14]. This is also visible from the intermittent H-bond autocorrelation function displayed in Fig. Supplementary8-b. Although the H-bond characteristic time recorded at zero field (violet curve) is extended by

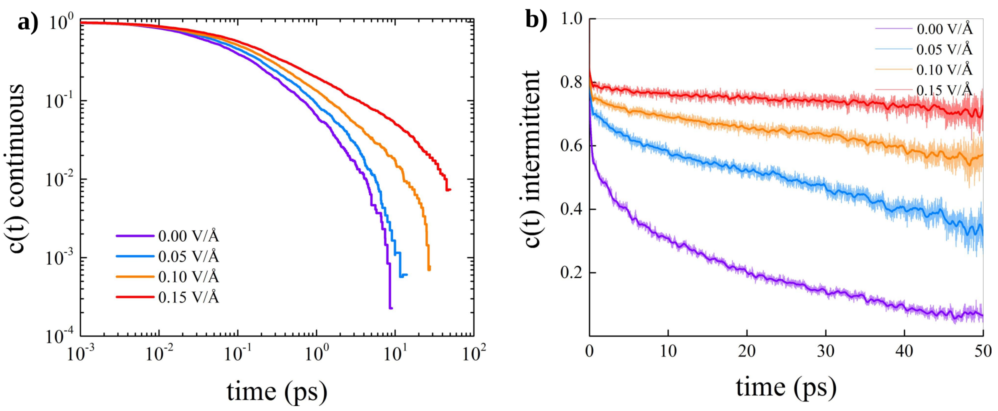

Fig. Supplementary 8 Log-log continuous (a) and linear-scale intermittent (b) H-bond autocorrelation functions calculated during the last 50 ps of the respective simulations.

the application of a field strength of 0.05 VÅ^{-1} (blue curve), a visible decay of the intermolecular correlations within the timescales of our simulations is recorded at the latter regime. Instead, a field of 0.10 VÅ^{-1} (orange curve) and a field of 0.15 VÅ^{-1} (red curve) clearly strengthen the H-bond persistence over sizably longer timescales. These results are fully consistent with the picture emerging from the partial Van Hove correlation functions shown in the main text (Fig. 2).

In Fig. Supplementary9 we report the oxygen-oxygen radial distribution function computed with a larger simulation box of 256 water molecules, at densities of 0.92 g·cm^{-3} and 0.95 g·cm^{-3} and at a temperature of 250 K (panels (a) and (b), respectively) for a sample without EF and a sample with a field of 0.15 VÅ^{-1}. These simulations reach $\sim$ 500 ps. It is possible to observe the development of an f-GW-like $g_{OO}(r)$ at both densities, indicating that the transition to f-GW reported in our work is not an artifact of small simulation boxes and that takes place for different densities.

In Fig. Supplementary10 we report $d = 4$, the percentage of four-folded water molecules at consecutive time windows. The blue stripe corresponds to the case of liquid water in the absence of EFs. In the presence of 0.05 VÅ^{-1} (red squares), $d = 4$ increases from $\sim$ 50% to $\sim$ 53% within the first 50 ps of the simulation, and keeps gradually increasing reaching a maximum of $\sim$ 56% in the last two time windows. Upon increasing the field to 0.10 VÅ^{-1} (green diamonds) we can observe that $d = 4$ computed within the first 50 ps is roughly the same as the case for 0.05 VÅ^{-1} computed on the same time window. On the other hand, the $d = 4$ linearly increases by $\sim$ 6% in the second and in the third time window. The $d = 4$ reaches then a plateau in correspondence with the last two time windows. Upon increasing the field strength to 0.15 VÅ^{-1}, we observe a sudden increase in the $d = 4$ to $\sim$ 56% within the first time window. Further increases occur at the later stages of the simulation as for the cases previously inspected with lower field strengths. It is worth noticing that the percentage of four-coordinated water molecules for 0.10 VÅ^{-1} and 0.15 VÅ^{-1} is almost indistinguishable towards the end of the simulation.

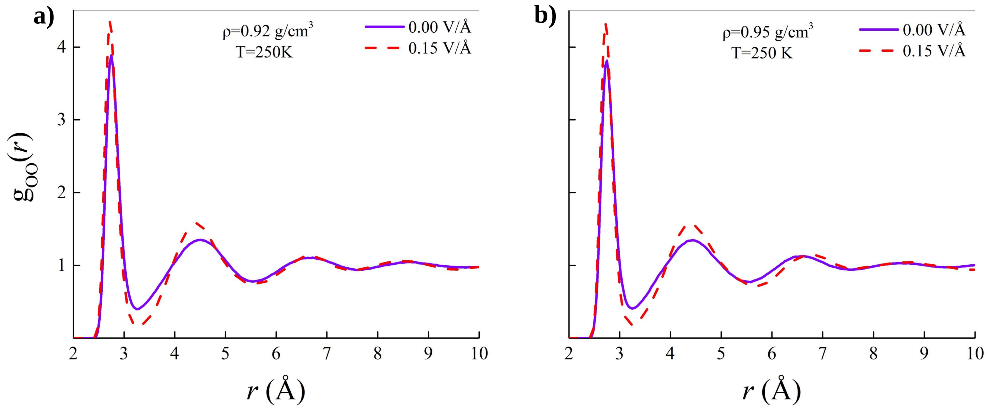

Fig. Supplementary 9 Oxygen-oxygen radial distribution functions in the absence of the field (violet solid lines) and in the presence of a field strength equal to 0.15 VÅ^{-1} from *ab initio* molecular dynamics simulations conducted on boxes containing 256 H_2O molecules in the supercooled regime (T = 250 K), and for densities of 0.92 g·cm^{-3} (a) and 0.95 g·cm^{-3} (b).

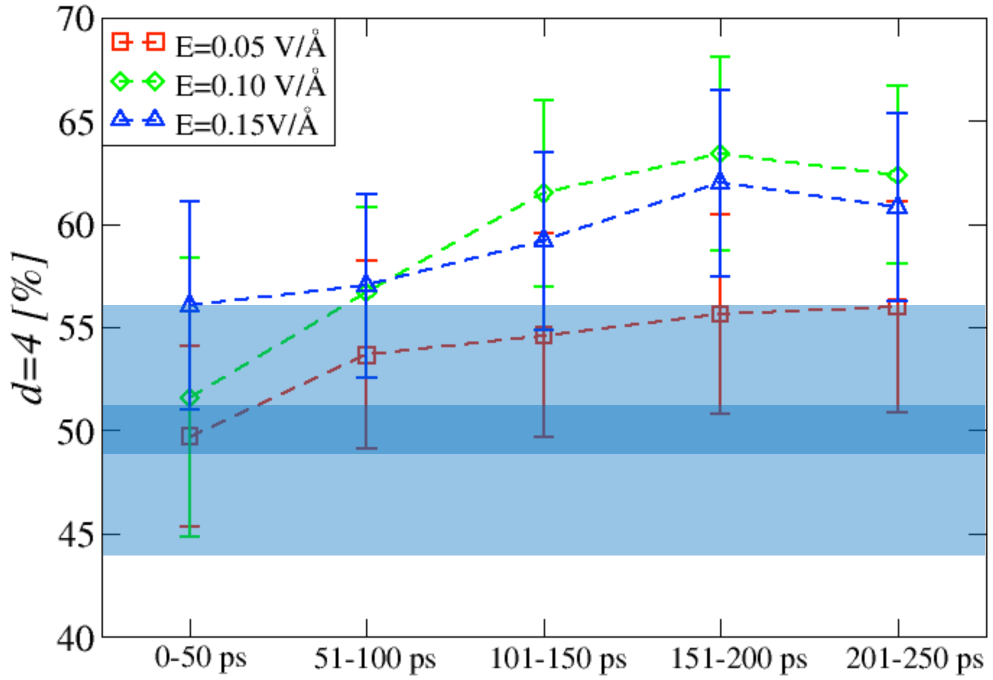

Fig. Supplementary 10 Percentage of four-coordinated water molecules computed on consecutive time windows and compared against the case of bulk water in the absence of EFs (blue stripe). Red circles refer to the case of 0.05 VÅ^{-1}, green diamonds to 0.10 VÅ^{-1}, blue triangles to 0.15 VÅ^{-1}.

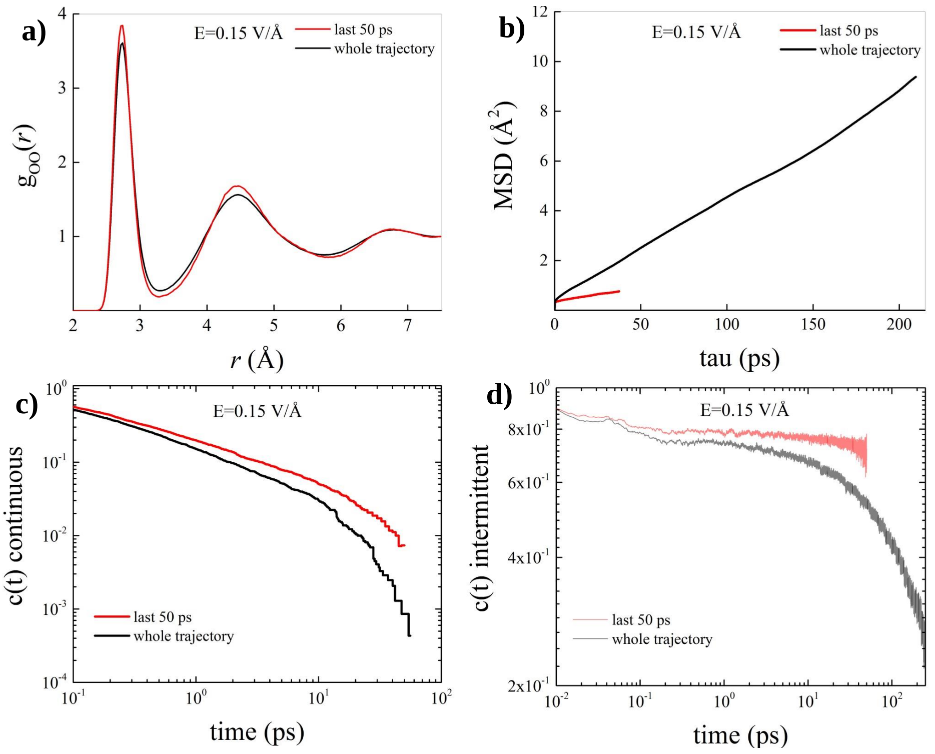

Fig. Supplementary 11 (a) Oxygen-oxygen radial distribution functions, (b) oxygen mean squared displacement, (c) log-log continuous and (d) intermittent autocorrelation functions of the H-bonds determined at an EF intensity of 0.15 VÅ^{-1} calculated over the whole trajectory (black lines) and during the last 50 ps of dynamics (red lines).

To further stress the relevance of the sampling at disjoint time windows, we report in Fig. Supplementary11 a series of structural and dynamical observables determined at the highest field intensity here explored (i.e., 0.15 VÅ^{-1}) and calculated over the whole 250-ps-long trajectory and on the last 50 ps of dynamics. The $g_{OO}(r)$ at 0.15 VÅ^{-1} determined over the whole trajectory exhibits smaller (higher) peaks (dips) with respect to the same quantity calculated over the last 50 ps of dynamics of the same trajectory, as displayed in Fig. Supplementary11-a. Interestingly, the importance of sampling at consecutive time frames is even more visible from dynamical rather than structural properties. In fact, to adequately evaluate the field effects on the translational degrees of freedom, the sampling at consecutive time windows here adopted appears to be necessary for the timescales affordable by *ab initio* simulations. As shown in Fig. Supplementary11-b, indeed, the mean squared displacement of the oxygen atoms determined over the whole trajectory at 0.15 VÅ^{-1} witnesses the mixing of different translational regimes, a circumstance leading to an underestimation of the EF-induced damping effect. This is not only true for translational but also for

rotational degrees of freedom, which are intimately related to the dynamics of the H-bond network. By direct comparison of the continuous (Fig. Supplementary11-c) and intermittent (Fig. Supplementary11-d) H-bond autocorrelation functions determined over different timescales (see legends), diverse H-bond characteristic times emerge. All these findings prove that relevant information on the effects produced by the application of external fields on liquid water can be unveiled only by accessing and isolating late portions of long *ab initio* simulations. Exclusively by adopting this strategy, it is possible to catch the *electrofreezing* effect induced by the field on the roto-translational degrees of freedom of water and, presumably, of other H-bonded systems.

To estimate the effects of EFs on the rotational dynamics, we have also computed the rotational autocorrelation functions $C_{rot}(t)$, that we report in Fig. S12 for the three fields and at consecutive time windows. Fig. S12 reports also the profile of $C_{rot}(t)$ computed for liquid water in the absence of EFs. The lowest field here inspected (upper panel) affects the rotational degrees of freedom mostly only after $\sim$ 50 ps. The profile of $C_{rot}(t)$ at higher times indicates that rotational degrees of freedom become increasingly slow but are always active, although very sluggish after $\sim$ 200 ps. Upon increasing the field strength to 0.10 VÅ^{-1} and to 0.15 VÅ^{-1} (middle and lower panel, respectively), molecular rotations become rapidly very slow even within the first 50 ps, this effect being more pronounced at the highest field. For both cases, the fields induce a complete freezing of molecular rotations after $150 - 200$ ps, as captured by the plateau of $C_{rot}(t)$.

In Fig. Supplementary 13 we report the difference between n-member rings at consecutive time windows τ and for the three fields here inspected. Such fluctuations do not disappear for 0.05 VÅ^{-1} because the system is in the liquid phase. On the other hand, we observe a flat-like profile for 0.10 VÅ^{-1} and 0.15 VÅ^{-1} occurring after $\sim$ 150 ps, i.e. in correspondence with the transition to f-GW.

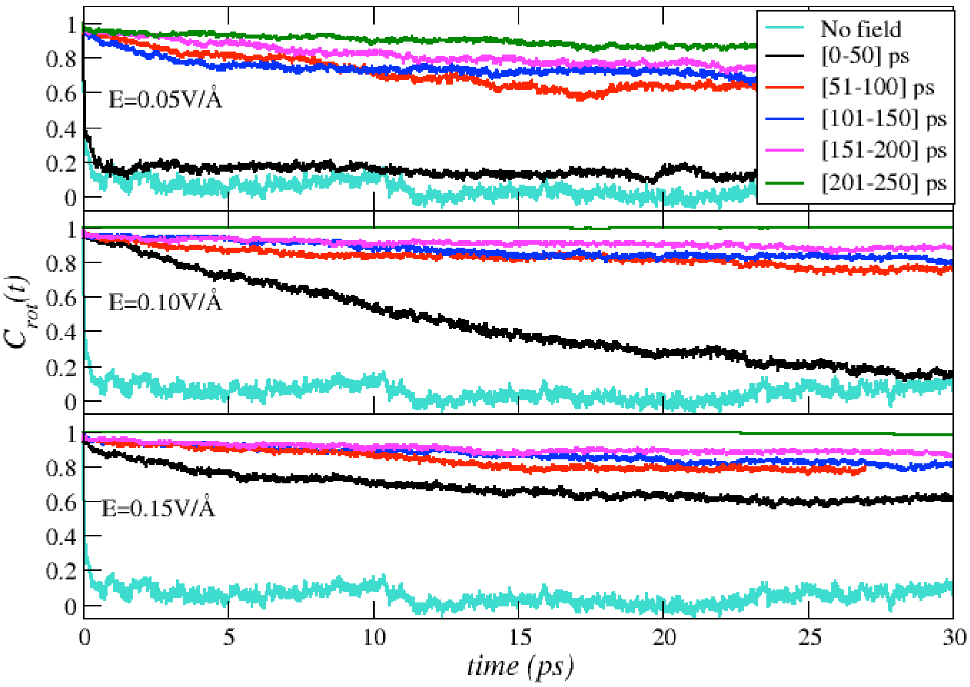

Fig. Supplementary 12 Rotational autocorrelation function at field intensities of 0.05 VÅ^{-1} (top), 0.10 VÅ^{-1} (middle), and 0.15 VÅ^{-1} (bottom) at different consecutive time windows (see legend). Each plot reports the autocorrelation function of liquid water for comparison.

References

[1] Brehm, M., Kirchner, B.: Travis - a free analyzer and visualizer for monte carlo and molecular dynamics trajectories. Journal of Chemical Information and Modeling **51**(8), 2007–2023 (2011)

[2] Thomas, M., Brehm, M., Fligg, R., Vöhringer, P., Kirchner, B.: Computing vibrational spectra from ab initio molecular dynamics. Phys. Chem. Chem. Phys. **15**, 6608–6622 (2013)

[3] Marzari, N., Vanderbilt, D.: Maximally localized generalized wannier functions for composite energy bands. Phys. Rev. B **56**, 12847–12865 (1997) https://doi.org/10.1103/PhysRevB.56.12847

[4] Marzari, N., Mostofi, A.A., Yates, J.R., Souza, I., Vanderbilt, D.: Maximally localized wannier functions: Theory and applications. Rev. Mod. Phys. **84**, 1419–1475 (2012) https://doi.org/10.1103/RevModPhys.84.1419

[5] Elgabarty, H., Kaliannan, N.K., Kühne, T.D.: Enhancement of the local asymmetry in the hydrogen bond network of liquid water by an ultrafast electric field

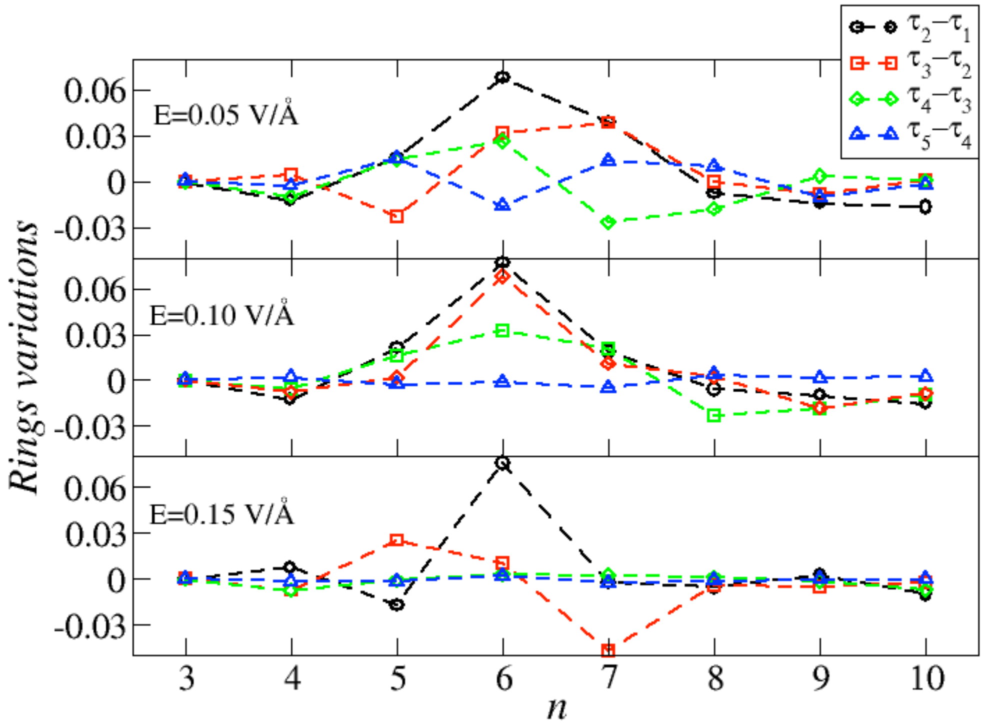

Fig. Supplementary 13 Difference between n-member rings between consecutive time windows τ. The upper panel refers to 0.05 VÅ^{-1}, the middle panel to 0.10 VÅ^{-1} and the lower panel to 0.15 VÅ^{-1}.

pulse. Scientific Reports **9**(1), 10002 (2019)

[6] Chau, P.L., Hardwick, A.J.: A new order parameter for tetrahedral configurations. Mol. Phys. **93**(3), 511–518 (1998)

[7] Errington, J.R., Debenedetti, P.G.: Relationship between structural order and the anomalies of liquid water. Nature **409**(6818), 318–321 (2001)

[8] Shiratani, E., Sasai, M.: Growth and collapse of structural patterns in the hydrogen bond network in liquid water. The Journal of chemical physics **104**(19), 7671–7680 (1996)

[9] Shiratani, E., Sasai, M.: Molecular scale precursor of the liquid–liquid phase transition of water. The Journal of chemical physics **108**(8), 3264–3276 (1998)

[10] Martelli, F., Torquato, S., Giovambattista, N., Car, R.: Large-scale structure and hyperuniformity of amorphous ices. Phys. Rev. Lett. **119**(13), 136002 (2017)

[11] Martelli, F.: Steady-like topology of the dynamical hydrogen bond network in

supercooled water. PNAS Nexus **1**(3), 090 (2022)

[12] Martelli, F., Giovambattista, N., Torquato, S., Car, R.: Searching for crystal-ice domains in amorphous ices. Phys. Rev. Mater. **2**(7), 075601 (2018)

[13] Formanek, M., Torquato, S., Car, R., Martelli, F.: Molecular rotations, multi-scale order, hyperuniformity, and signatures of metastability during the compression/decompression cycles of amorphous ices. J. Phys. Chem. B **127**(17), 3946–3957 (2023)

[14] Han, S., Kumar, P., Stanley, H.E.: Hydrogen-bond dynamics of water in a quasi-two-dimensional hydrophobic nanopore slit. Phys. Rev. E **79**, 041202 (2009) https://doi.org/10.1103/PhysRevE.79.041202
